# Supplementary material for: Seed Size, Seed Dispersal Traits, and Plant Dispersion Patterns for Native and Introduced Grassland Plants
Source: Plants (Basel). 2023 Feb 24;12(5):1032. doi: 10.3390/plants12051032 (PMC10005497; doi:10.3390/plants12051032)
Supplement: Supplementary file 1 [file plants-12-01032-s001.zip › plants-2213896-supplementary.pdf]

Table S1. Results of GLMs evaluating relationships between plant dispersion and each of two seed traits, seed mass and the presence of seed dispersal traits, for 23 native and 25 introduced grasslands species in Montana, USA. Dispersion was measured and analyzed at either the local scale as the proportion of plots occupied per site or at the broad scale as the proportion of sites occupied. For all models, df = 1 and 44.

	Seed Mass				Dispersal Structures			
	Local Scale		Broad Scale		Local Scale		Broad Scale	
	F	*p*	*F*	*p*	*F*	*p*	*F*	*p*
Trait	1.7	0.1991	1.1	0.2948	2.1	0.1591	1.9	0.1723
Origin	<0.1	0.9198	0.1	0.7167	2.5	0.1208	0.8	0.3862
Trait x Origin	3.5	0.0673	2.0	0.1647	0.5	0.4922	0.6	0.4575

Table S2. Seed traits evaluated for 23 native (N) and 25 introduced (I) grassland plant species in Montana, USA, in order of their measured seed mass (averaged over the number of samples indicated in parentheses), which was used to divide seeds into small (S) and large (L) seed size classes for analysis based on a natural break in the data. Also given is the seed mass value obtained from the TRY trait database (averaged over the number of records indicated in parentheses); note that the database did not contain data for four species (indicated by a dash). Seed mass was compared between data sources using a one sample *t*-test.

Species	Origin	Seed Size	Disp. Struct.	Seed Measures				Comparison to TRY		
				Length (mm)	Width (mm)	SA (mm2)	Mass (mg)	Mass (mg)	$t^{\dagger}$	*p*
Lithophragma parviflorum	N	S	None	0.48	0.27	0.10	0.012 (9)	–	–	–
Draba verna	I	S	None	0.42	0.28	0.10	0.016 (9)	–	–	–
Filago arvensis	I	S	Pappus	0.84	0.31	0.22	0.028 (9)	0.400 (1)	649.0	<0.0001
Sisymbrium loeselii	I	S	None	0.81	0.45	0.33	0.052 (9)	0.109 (5)	22.0	<0.0001
Holosteum umbellatum	I	S	None	0.71	0.51	0.32	0.065 (9)	0.115 (2)	28.7	<0.0001
Arenaria serpyllifolia	I	S	None	0.61	0.45	0.29	0.067 (9)	0.077 (16)	8.2	<0.0001
Veronica verna	I	S	None	0.90	0.64	0.89	0.070 (9)	0.108 (7)	21.6	<0.0001
Achillea millefolium	N	S	Wing	1.50	0.45	0.36	0.073 (9)	0.197 (26)	47.4	<0.0001
Boechera retrofracta	N	S	Wing	1.02	0.88	0.67	0.097 (9)	0.126 (4)	5.6	0.0005
Hypericum perforatum	I	S	None	1.09	0.45	0.45	0.098 (9)	0.277(17)	36.0	<0.0001
Verbascum blattaria	I	S	None	0.79	0.54	0.39	0.109 (9)	0.108 (3)	0.5	0.6246
Linaria dalmatica	I	S	None	1.47	0.95	1.04	0.134 (9)	0.140 (1)	0.8	0.4479
Myosotis stricta	I	S	None	1.03	0.64	0.52	0.147 (9)	0.113 (1)	5.0	0.0011
Apera interrupta	I	S	Awn	2.04	0.39	0.83	0.151 (9)	0.095 (2)	7.5	<0.0001
Collinsia parviflora	N	S	None	1.68	0.98	1.38	0.151 (9)	1.451 (7)	70.5	<0.0001
Sisymbrium altissimum	I	S	None	0.91	0.58	0.50	0.177 (12)	0.190 (9)	1.1	0.3005
Potentilla recta	I	S	None	1.12	0.79	0.73	0.198 (9)	0.229 (6)	4.0	0.0039
Poa compressa	I	S	None	2.37	0.63	1.30	0.231 (9)	0.219 (10)	1.2	0.2736
Koeleria macrantha	N	S	Awn	4.77	0.92	3.97	0.263 (9)	2.458 (15)	24.0	<0.0001
Poa secunda	N	S	None	3.83	0.76	2.37	0.317 (9)	0.325 (3)	0.0	0.9992
Rumex acetosella	I	S	None	1.54	0.96	1.04	0.333 (9)	0.695 (30)	18.3	<0.0001
Arenaria congesta	N	S	None	1.64	0.99	1.23	0.336 (9)	0.640 (1)	21.2	<0.0001
Camelina microcarpa	I	S	None	1.21	0.84	0.84	0.347 (9)	0.294 (5)	7.0	0.0001
Festuca idahoensis	N	S	Awn	5.57	0.81	4.63	0.441 (9)	1.037 (3)	36.0	<0.0001
Taraxacum officinale	I	S	Pappus	3.42	0.69	1.86	0.450 (9)	–	–	–

Alyssum alyssoides	I	S	None	1.56	1.18	1.53	0.561 (9)	0.690 (6)	7.9	<0.0001
Lactuca serriola	I	S	Pappus	3.40	0.80	1.96	0.572 (9)	0.464 (13)	3.6	0.0074
Heterotheca villosa	N	S	Pappus	2.81	0.87	1.23	0.693 (9)	0.769 (3)	1.9	0.0951
Plantago patagonica	N	S	None	2.01	1.00	1.67	0.734 (9)	0.542 (3)	3.9	0.0024
Microsteris gracilis	N	S	None	2.35	1.49	2.71	1.069 (9)	1.111 (7)	1.1	0.2965
Geum triflorum	N	S	Plume	2.79	0.84	1.19	1.291 (9)	0.949 (5)	11.7	<0.0001
Zigadenus venenosus	N	L	None	5.39	1.47	6.01	1.667 (9)	–	–	–
Eriogonum umbellatum	N	L	None	4.69	1.45	4.17	1.692 (9)	2.308 (6)	14.0	<0.0001
Danthonia unispicata	N	L	Awn	7.46	1.56	9.92	1.994 (9)	1.132 (1)	12.9	<0.0001
Centaurea stoebe	I	L	Pappus	2.91	1.32	4.11	2.001 (9)	2.058 (3)	1.1	0.2954
Gaillardia aristata	N	L	Pappus	2.80	1.56	2.20	2.044 (9)	22.532 (4)	215.4	<0.0001
Fritillaria pudica	N	L	None	5.55	4.44	17.95	2.297 (9)	2.336 (3)	0.4	0.727
Erodium cicutarium	I	L	Awn	4.32	1.01	5.51	2.499 (9)	1.985 (18)	14.3	<0.0001
Euphorbia virgata	I	L	None	2.28	1.53	2.94	2.909 (9)	2.876 (8)	1.4	<0.0001
Bromus japonicus	I	L	Awn	6.03	1.55	8.29	2.962 (12)	2.577 (5)	7.1	<0.0001
Elymus spicatus	N	L	Awn	8.40	1.13	4.73	3.662 (9)	4.040 (3)	9.7	<0.0001
Bromus tectorum	I	L	Awn	8.46	0.79	8.03	3.690 (12)	2.808 (19)	10.7	<0.0001
Lomatium triternatum	N	L	Wing	7.95	4.72	11.08	4.520 (9)	2365.908 (3)	205.0	<0.0001
Balsamorhiza sagittata	N	L	None	7.77	1.94	12.56	4.702 (9)	5.304 (4)	3.3	0.0115
Tragopogon dubius	I	L	Pappus	10.39	1.27	6.60	7.172 (9)	8.924 (10)	4.1	0.0036
Stipa comata	N	L	Awn	9.41	1.09	5.20	7.568 (9)	5.655 (6)	13.5	<0.0001
Lupinus sericeus	N	L	None	4.56	3.40	13.18	20.378 (12)	20.349 (2)	0.1	0.9647
Lithospermum ruderale	N	L	None	4.33	3.40	11.23	27.162 (12)	17.345 (3)	17.5	<0.0001

† df = 8 except in five cases (*B. japonicus*, *B. tectorum*, *Filago arvensis*, *L. ruderale*, and *L. sericeus*) where df = 11 (due to the extra sample weighed per population).

Supplemental Materials S1. Seed mass patterns obtained when original measures were replaced with estimates from the TRY trait database.

We repeated analyses involving seed mass while replacing our measured values of this trait with those obtained from the TRY trait database. Despite the fact that seed mass differed substantially between sources in many cases (see Results and Table S2), analyses revealed parallel patterns. Specifically, we once again saw a linkage among seed dispersal structures, species origin, and seed mass (now represented by estimates from the TRY database). The presence of seed dispersal structures tended to vary with both seed mass ($F_{1,40} = 4.9$, $p = 0.034$) and species origin (seed mass x origin: $F_{1,40} = 4.9$, $p = 0.033$). As seen in our original analysis, seed mass was positively correlated with the presence of dispersal structures for introduced and not native taxa. Similarly, 83% of 6 exotics with large seeds (> 1.5 mg) had dispersal structures compared to only 18% of 17 smaller-seeded exotics ($p = 0.009$), while the proportion of native species with these structures did not differ between large-seed (50% of 10 species) and small-seed (55% of 11 species) classes ($p = 1.0$). Origin significantly affected the presence of dispersal structures ($F_{1,40} = 4.5$, $p = 0.04$), but in alignment with original results, the pattern varied with seed mass as indicated by the significant interaction between origin and seed mass (test statistics above); introduced species were generally more likely than natives to have seed dispersal structures at large but not small seed masses.

Seed mass, as represented by estimates from the TRY trait database, was also associated with plant dispersion. Local-scale dispersion (proportion of plots occupied per site) again tended to correlate positively with seed mass for introduced but not native taxa, as supported by a marginally significant interaction between seed mass and origin ($F_{1,40} = 3.1$, $p = 0.089$). Consistent with the original analysis, the seed mass relationship did not vary significantly with origin when we considered broad-scale dispersion (proportion of sites occupied; $F_{1,40} = 2.5$, p = 0.12).
